# Emerging Marine Biotoxins

**DOI:** 10.3390/toxins11060314

**Published:** 2019-06-03

**Authors:** Arjen Gerssen, Ana Gago-Martínez

**Affiliations:** 1 Wageningen Food Safety Research (WFSR), Wageningen University & Research, Akkermaalsbos 2, 6708WB Wageningen, The Netherlands; arjen.gerssen@wur.nl; 2 Department of Analytical and Food Chemistry, University of Vigo, Campus Universitario de Vigo, 36310 Vigo, Spain; 3 European Union Reference Laboratory for Marine Biotoxins, CITEXVI, Campus Universitario de Vigo, 36310 Vigo, Spain

The emergence of marine biotoxins in geographical areas where they have never been reported before is a concern of considerable impact on seafood contamination, and consequently, on public health. Several groups of marine biotoxins, in particular tetrodotoxins, ciguatoxins, and palytoxins, are included among the relevant marine biotoxins that have recently emerged in several coastal areas. A similar situation has been observed in freshwater, where cyanobacterial toxins such as microcystins might end up in unexpected areas such as the estuaries where shellfish are cultivated. Climate change and the increased availability of nutrients have been considered as the key factors in the expansion of all of these toxins into new areas; however, this could also be due to more intense biological invasions, more sensitive analytical methods, or perhaps even an increased scientific interest in these natural contaminations. The incidences of human intoxications due to the consumption of seafood contaminated with these toxins have made their study an important task to accomplish, in order to protect human health. This special issue has a focus on a wide variety of emerging biotoxin classes.

One of the most important classes of emerging toxins are ciguatoxins, which until recently were considered endemic to (sub)tropical regions in the Pacific and Indian Ocean as well as the Caribbean Sea, but are nowadays responsible for intoxications in other places worldwide. This is particularly so in Europe, not only through the consumption of imported ciguatoxin contaminated seafood from endemic areas, but also because of their occurrence in particular areas of Macaronesia such as Azores, Madeira Islands (Portugal) and the Canary Islands (Spain). Ciguatoxins are produced by the *Gambierdiscus* species. The effect of microbiological factors on both growth and toxin production is still poorly understood. Wang et al. investigated the effect of quorum sensing bacteria on both of these parameters [1]. It was found that quorum sensing bacteria have a major influence on the growth as well as the toxin production. This indicates the difficulty of predicting the growth and toxin production in environmental conditions as many factors have an influence on these algal toxins. Ciguatoxins accumulate in higher trophic fish; if these fish are consumed, this might lead to intoxication. To confirm the presence of these toxins in fish, confirmatory methods that use mass spectrometric detection can be used. However, these methods are challenging, mainly due to the lack of commercially available reference materials. A combination of toxicity screening with an in vitro cell test, i.e., the neuroblastoma cell viability test, is often used to screen the samples. This assay can also be used for effect directed analysis as that conducted by Estevez et al. [2]. Sample extracts from fish collected from Macaronesia were fractionated. The fractions were screened for toxicity and the active fractions were analyzed by liquid chromatography and tandem mass spectrometry (LC-MS/MS) to confirm the ciguatoxins involved in these contaminations. Caribbean ciguatoxin-1 (C-CTX-1) has been identified as being mainly responsible for the CTX contamination in these areas, although some other C-CTX1 analogues and metabolites have also been identified. Reis Costa et al. also reported on the presence of C-CTX1 in fish from the Portuguese area of Madeira and the Selvagem Islands [3]. It is difficult to conclude if ciguatoxins are really new to Macaronesia, or if their recent identification has been due to the increased research and knowledge on this particular issue. Alternative approaches can be also used for screening of a wide variety of toxins, based on non-targeted high resolution mass spectrometry. However, the confirmation of the suspected toxins by their accurate mass is still challenging, either due to the lack of certified standards or the lack of sensitivity of the high resolution mass spectrometer. Despite these limitations, significant improvements have been made [4]. In addition, the sensitivity of the MS detection of polytoxins has been considerably increased by using the so called cationization [5].

Comparable to the ciguatoxins is the occurrence of tetrodotoxin (TTX), which was recently associated with contaminations in bivalves, and not only to fish and gastropods as reported in the past. Furthermore, it was not expected to occur in temperate water conditions. However, researchers have demonstrated its presence in shellfish from the UK as well as seasonal occurrence in the Netherlands [6,7]. In the Dutch situation, an action limit was set at 44 µg TTX/kg shellfish, which is based on the opinion by European Food Safety [8]. As TTX has the same mode of action as saxitoxin (STX), Finch et al. investigated the acute toxicity of TTX and STX/TTX mixtures in mice through various routes of administration [9]. They found additivity between STX and TTX, and therefore concluded that TTX could be treated as a member of the paralytic shellfish toxin group (i.e., STXs).

Furthermore, not truly marine biotoxins, cyanobacterial toxins such as microcystins are known to cause problems in drinking water and might not only end up in shellfish and fish produced in freshwater conditions, but also in estuaries [10]. The toxicological effects of non-proteinogenic amino acid beta-methyl-amino-L-alanine (BMAA), a neurotoxin produced by cyanobacteria, on the putative neurodegeneration of newly identified specific dopaminergic neurons in the optic ganglia/brain complex of *D. magna* have been evaluated by using quantitative tyrosine-hydroxylase immunohistochemistry and fluorescence cytometry [11]. Interesting information on the physiological properties of cyanobacteria phylogenetically related, but living in different environments, has been characterized by microscopy, molecular, and toxicity analyses. A variable pattern of toxicity was exhibited, in accordance with the constraints imposed by the host environments [12]. In general, based on more intensive agriculture, more nutrients from fertilizers might end up in surface waters, which improve the conditions for the growth of various cyanobacteria that might eventually threaten our food safety.

This special issue also contains original information on novel sequencing approaches to identify natural toxins. These include short sequence tags that can be used to elucidate full-length peptide sequences to identify natural toxins like conotoxins from the venom of the cone snail Conus geographus by NMR spectroscopy [13], or the use of novel sequencing approaches to identify a diversity of toxin-related peptide sequences by means of computational processing, comprising of structural phylogenetic analysis, model prediction, and the dynamics simulation of peptide–receptor interaction [14]. Sequencing approaches have been also used to characterize the whole-genome of Chinese Yellow Catfish, providing a valuable genetic resource for high-throughput identification of toxin genes [15].

To summarize, this special issue contains original contributions that allow for significant advance on the knowledge of a wide variety of emerging marine biotoxin classes as well as technological developments to screen and detect or even evaluate the toxicological effects of these toxins in various matrices and environments.
